# A Dynamic Convolution Kernel Generation Method Based on Regularized Pattern for Image Super-Resolution

**DOI:** 10.3390/s22114231

**Published:** 2022-06-01

**Authors:** Hesen Feng, Lihong Ma, Jing Tian

**Affiliations:** 1 School of Electronics & Information Engineering, South China University of Technology, Guangzhou 510640, China; 201920111160@mail.scut.edu.cn; 2 National Research Center for Mobile Ultrasonic Detection, Guangzhou 510640, China; 3 Institute of Systems Science, National University of Singapore, Singapore 119615, Singapore; tianjing@nus.edu.sg

**Keywords:** image super-resolution, dynamic convolution kernel, regularized pattern, multi-task learning, RPB-RDN

## Abstract

Image super-resolution aims to reconstruct a high-resolution image from its low-resolution counterparts. Conventional image super-resolution approaches share the same spatial convolution kernel for the whole image in the upscaling modules, which neglect the specificity of content information in different positions of the image. In view of this, this paper proposes a regularized pattern method to represent spatially variant structural features in an image and further exploits a dynamic convolution kernel generation method to match the regularized pattern and improve image reconstruction performance. To be more specific, first, the proposed approach extracts features from low-resolution images using a self-organizing feature mapping network to construct regularized patterns (RP), which describe different contents at different locations. Second, the meta-learning mechanism based on the regularized pattern predicts the weights of the convolution kernels that match the regularized pattern for each different location; therefore, it generates different upscaling functions for images with different content. Extensive experiments are conducted using the benchmark datasets Set5, Set14, B100, Urban100, and Manga109 to demonstrate that the proposed approach outperforms the state-of-the-art super-resolution approaches in terms of both PSNR and SSIM performance.

## 1. Introduction

The goal of *single image super-resolution* (SISR) is to reconstruct high-quality *high-resolution* (HR) images from degraded *low-resolution* (LR) images. It has very wide applications in video surveillance, remote sensing, and medical and military imaging. Another interesting work related to SISR is the face hallucination which enlarges input regions by approximately linear mapping SVD values among different resolutions [1]. Its hallucination capability was further expanded with the same mapping across different views [2]. The pioneering networked SISR work was done by Dong et al. [3]. Their proposed neural network SRCNN established an end-to-end mapping from an input interpolated LR image to the output HR image. Then VDSR [4], DRCN [5], DRRN [6], and MemNet [7] were successively proposed, which further improved the image reconstruction performance. These methods up-sampled an LR input at the very first to the required size of a network output, rather than using an upscaling module to increase the spatial resolution at the end.

However, recent research works found that such an early interpolation on LR image will inevitably result in detail loss and greatly increase the amount of model calculation. Extracting features from the original LR input and increasing the spatial resolution at the end of the network has become a popular deep SISR structure. Shi et al. proposed an efficient sub-pixel convolution layer in ESPCN [8], which enlarged the LR feature map to the output size at the end of a network. With the efficient sub-pixel convolution layer, many methods, such as EDSR [9], RDN [10], RFANet [11], SAN [12], DID [13], treated SR recovery with different scale factors as independent tasks, and applied sub-pixel convolution layers for feature map expansion at the end. While sub-pixel convolutional layers are only feasible to integer scale factors, and a specific network model must be designed for each scale factor, each network model can magnify images merely with a fixed integer scale factor.

To avoid the design of different network models for different scale factors, the meta-learning technique [14] has been introduced to develop various SR approaches. The feed-forward model (FFM) in the meta feature representation [14] provided a feedforward mapping method that directly predicted the required parameters of a test instance. Similar to the Hypernetworks [15], the weight of another neural network was generated in a feedforward process. To perform image super-resolution at any scale in one model, Hu et al. proposed Meta-SR [16] to use the Meta-Upscale Module to improve the spatial resolution at the end of the network. For different scale factors and position coordinate offsets, the weight prediction network in the Meta-Upscale Module can generate different convolution kernels to generate the final SR image. However, the Meta-Upscale Module still shared the convolution kernel spatially and did not consider the content information of the current image. Chen et al. proposed LIIF [17], using a multi-layer perceptron at the end of the network to replace the traditional upscaling layer and predict the gray value of each pixel in the output SR image. However, since the input of the multi-layer perceptron is a one-dimensional vector, the original position information of the feature vector will be lost in the process of converting a multi-dimensional vector of the feature map into a one-dimensional vector of an input.

The major challenge of single image SR is how to perform upscaling reconstruction adaptively to the spatially variant image content. According to the characteristics of involution [18], if the convolution kernel is shared spatially, the parameters of the convolution kernel cannot be flexibly adjusted to match different inputs. On the contrary, we can use space-specific kernels for more flexible modeling in the spatial dimension. Similar to the space-specific involution, introducing a regularized pattern to guide the generation of convolution kernel will be helpful in the upscaling module. Motivated by this, in this paper, we propose a specific regularized pattern extraction network to extract the regularized pattern from LR features and then generate a space-specific convolution kernel according to different regularized patterns.

The two contributions of this paper are summarized as follows.

(1) A regularized pattern extraction method is proposed to extract the regularized pattern from LR features. This will adaptively guide the image reconstruction in a spatially variant manner. Furthermore, both position information and scale information are used in the weight prediction network with the proposed regularized pattern. As a result, the convolution weight prediction network can accurately match the relationship between input parameters and output convolution kernel parameters.

(2) A dynamic convolution kernel generation method is proposed to generate the most matching convolution kernel parameters according to the regularized pattern and position and scale information of the current position. Consequently, the pixels at different positions in the SR image can be processed differently, which enhances the texture consistency with the HR image and improves the network performance. 

The rest of this paper is organized as follows. The dynamic convolution kernel generation method is proposed and then further exploited to develop a super-resolution approach in Section 2. The proposed approach is evaluated with state-of-the-art approaches in extensive experiments in Section 3. Finally, Section 4 concludes this paper.

## 2. Proposed Dynamic Convolution Kernel Generation Based on Regularized Pattern for Image Super-Resolution

**Figure 1:**
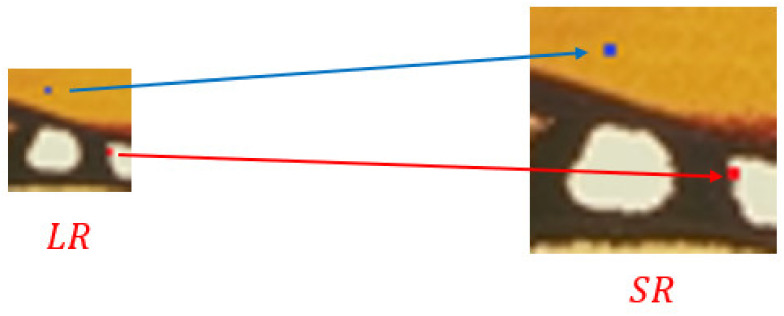
A conceptual illustration of our proposed SR method. Pixels at different positions are upsampled using different convolution kernels that match the regularized pattern of the current position.

Different pixel points in the LR image have different image contents. As shown in Figure 1, the blue points are in the flat color block area, and the red points are in the edge area. During the Meta-SR upsampling process [16], the convolution kernels used for these two positions are the same. The difference in the content information of these two positions is not considered. We propose a dynamic convolution kernel generation method to adaptively generate convolution kernels according to local image content, which is represented by using the proposed regularized pattern. For the blue and red points in Figure 1, the proposed method produces different convolution kernels matching their regularized content patterns, implementing space-specific reconstruction operations.

Assuming that an input LR image is $I^{LR}$, the LR feature $F^{LR}$ is extracted from $I^{LR}$ by the LR feature extraction network. We use the feature tensor $V\in R^{H\times W\times inC}$ to represent the $F^{LR}$, where H is the height of $I^{LR}$, W is the width of $I^{LR}$, and inC is the number of channels of V. In the feature tensor V, the feature vector $V_{i^{\prime },j^{\prime }}\in R^{inC}$ corresponds to the feature representation on the pixel point $\left(i^{\prime },j^{\prime }\right)$ of the LR image.

### 2.1. Proposed Regularized Pattern Extraction Method

The regularized pattern extraction method is proposed in this section to guide the image upscaling reconstruction. Different pixel positions on the input LR image $I^{LR}$ contain different image content, such as relatively smooth background regions, or edges of an object that changes drastically. Their differences are manifested in their features $F^{LR}$. We define the regularized pattern P as

(1) $$P=p\left(F^{LR}\right)=S\left(\sigma \left(F^{LR}⊗W_{1}+B_{1}\right)⊗W_{2}+B_{2}\right)$$

where p() is the regularized pattern extraction function, ⊗ is the convolution operator, $W_{1}$ and $B_{1}$ are the weight and bias of the first convolution layer on regularized pattern extraction network, $W_{2}$ and $B_{2}$ are the weight and bias of the second convolution layer on regularized pattern extraction network, σ() is the Relu activation function, S() is the Sigmod activation function.

The regularized pattern defined in (1) is an abstraction of the LR feature $F^{LR}$, features that can distinguish content information of different positions are merged to obtain the regularized patterns. During the model training, the weight of the convolution kernel in the regularized pattern extraction network defined in (1) is constantly updated under the constraint of the L1 loss function, focusing on features that can best distinguish the content information to seek the regularized pattern vector with the least structural risk.

### 2.2. Proposed Dynamic Convolution Kernel Generation Method

In this section, a dynamic convolution kernel generation method is proposed to adaptively generate convolution kernels according to local image content, which is represented using the proposed regularized pattern described in Section 2.1.

First, task-level samples and data samples need to be generated. Suppose that the scale factor range is $\left[r_{min},r_{max}\right]$ when performing SR reconstruction on LR images, and the probability of using each scale factor in the range for super-resolution reconstruction is equal, that is, the distribution $p\left(r\right)$ of the scale factor r is a discrete uniform distribution in $\left[r_{min},r_{max}\right]$ as

(2) $$p\left(r\right)=U\left(r_{min},r_{max}\right)$$

We use the values of all scale factors in the distribution (2) to downsample the training set HR images to obtain the training set LR images corresponding to different scale factors r. Each time a scale factor $r_{s}$ is randomly selected from the distribution $p\left(r\right)$ as the current task, and then a pair of LR-HR image patches are randomly selected from the training set corresponding to the scale factor $r_{s}$ as training samples.

Suppose that the length and width of the LR image patch are L, there are $L^{2}$ pixels on the LR image patch, and there are ${\left(⎣L\times r_{s}⎦\right)}^{2}$ pixels on the corresponding reconstructed SR image patch. The weight prediction network needs to generate a convolution kernel for each pixel in the SR image matching its RP, position, and scale information. Then the generated convolution kernels are used to map the LR image to the HR image. So, the number of data samples in the current task with factor $r_{s}$ is ${\left(⎣L\times r_{s}⎦\right)}^{2}$.

Second, given a scale factor $r_{s}$, the input LR image $I^{LR}$ with the height L and the width W, the LR feature obtained after passing $I^{LR}$ through the feature extraction network is $F^{LR}$. Then, $F^{LR}$ is highly abstracted to extract the regularized pattern P, which represents the structure of different position information to distinguish image content at different locations as

(3) $$P=p_{\alpha }\left(F^{LR}\right)$$

where $p_{\alpha }$ is the regularized pattern extraction function and α is the parameter of the regularized pattern extraction network.

Third, for a pixel point $\left(i,j\right)$ in the SR image, the mapping pixel position in the LR image is $i^{\prime },j^{\prime }$, and the position and scale information is $M_{i,j}$ which can be obtained as follows. Suppose that for the pixel $\left(i,j\right)$ in $I^{SR}$, its mapping $\left(i^{\prime },j^{\prime }\right)$ can always be found in $F^{LR}$, where the $V_{i^{\prime },j^{\prime }}$ is most closely related to the RGB value of the pixel $\left(i,j\right)$ in $I^{SR}$. The mapping formula from $I^{SR}$ to $F^{LR}$ is as [16]

(4) $$i^{\prime },j^{\prime }=m\left(i,j\right)=(\left\lfloor \frac{i}{r}\rfloor ,\lfloor \frac{j}{r}\right\rfloor )$$

where m() is the position mapping function, r is the scale factor, and ⎣ ⎦ is the floor function. Then, for the feature vector $V_{i^{\prime },j^{\prime }}$ in the LR feature $F^{LR}$, the corresponding multiple pixel points $\left(i,j\right)$ in $I^{SR}$ have a different relative positional relationship with $V_{i^{\prime },j^{\prime }}$. Define the relative offset function to express this difference [16]

(5) $$o\left(i\right)=(\frac{i}{r}-\lfloor \frac{i}{r}\rfloor )$$

where o() is the relative offset function. Then, the position and scale information $M_{i,j}$ at the pixel point $\left(i,j\right)$ of the SR image $I^{SR}$ can be obtained as [16]

(6) $$M_{i,j}=\left(o\left(i\right),o\left(j\right),\frac{1}{r}\right)=\left(\left(\frac{i}{r}-\lfloor \frac{i}{r}\rfloor \right),\left(\frac{j}{r}-\lfloor \frac{j}{r}\rfloor \right),\frac{1}{r}\right)$$

Fourth, the corresponding regularized pattern is the vector $P_{i^{\prime },j^{\prime }}$ in position $i^{\prime },j^{\prime }$ of P. For different pixels, the regularized pattern, location, and scale information are different. That means the relative deviation from its mapped location and the structural information of the location are unique. We generate the best-matched convolution weights for each pixel as

(7) $$W_{i,j}=F_{\theta }\left(P_{i^{\prime },j^{\prime }},M_{i,j}\right)$$

where $F_{\theta }$ is the convolution weight prediction function, θ is the parameter of the convolution weight prediction network, and $W_{i,j}$ is the convolution weight corresponding to the pixel $\left(i,j\right)$ in the SR image. The convolution weight prediction network generates a total of (L×r×W×r) convolutions to form the convolution weight set $W_{set}$ as

(8) $$W_{set}=F_{\theta }\left(P,M\right)$$

Fifth, for the gray value of the pixel point $\left(i,j\right)$ in the SR image, the LR feature $F_{i^{\prime },j^{\prime }}^{LR}$ at the mapping position $i^{\prime },j^{\prime }$ in the LR image is the most closely related. Performing matrix product of the convolution weight $W_{i,j}$ and the LR feature $F_{i^{\prime },j^{\prime }}^{LR}$ we obtain the gray value $V_{i,j}$ of the pixel point as

(9) $$V_{i,j}=F_{i^{\prime },j^{\prime }}^{LR}W_{i,j}$$

For the entire SR image, it is obtained by upsampling the LR features as

(10) $$I^{SR}=f_{W_{set}}\left(F^{LR}\right)=f_{F_{\theta }\left(p_{\alpha }\left(F^{LR}\right),M\right)}\left(F^{LR}\right)$$

where $f_{W_{set}}$ is the upsampling function, and $W_{set}$ is the convolution kernel weight set.

**Figure 2:**
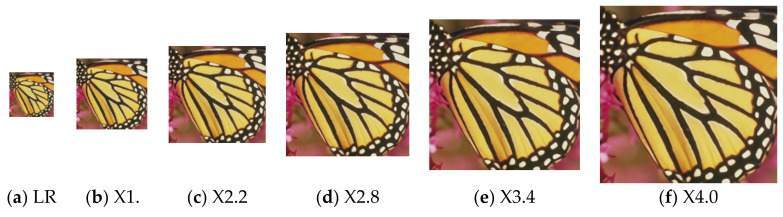
SR images generated by the proposed method at multiple scales.

Sixth, for the generated SR image patch, the L1 loss function is used to measure the error between the SR image patch and the HR image patch
  (11) $$L_{s}=\sum \left|I^{SR}-I^{HR}\right|=\sum |f_{F_{\theta }\left(p_{\alpha }\left(F^{LR}\right),M\right)}\left(F^{LR}\right)-I^{HR}|$$
  where $L_{s}$ is the error between $I^{SR}$ and $I^{HR}$ in current task with the scale factor $r_{s}$. In each task, the regularized pattern extraction network parameters α and convolution weight prediction network parameters θ are updated using gradient descent:
  (12) $$\alpha^{\prime }=\alpha -\beta \nabla_{\alpha }L_{s}=\alpha -\beta \nabla_{\alpha }\sum |f_{F_{\theta }\left(p_{\alpha }\left(F^{LR}\right),M\right)}\left(F^{LR}\right)-I^{HR}|$$
  (13) $$\theta^{\prime }=\theta -\beta \nabla_{\theta }L_{s}=\theta -\beta \nabla_{\theta }\sum |f_{F_{\theta }\left(p_{\alpha }\left(F^{LR}\right),M\right)}\left(F^{LR}\right)-I^{HR}|$$
  where α and θ are the parameters before the update, $\alpha^{\prime }$ and $\theta^{\prime }$ are the parameters after the update, and β is the learning rate.
By continuously extracting different scale factors from the distribution as different tasks to train the model, the parameters α and θ are continuously updated. The purpose of meta-learning training is to obtain appropriate parameters α and θ, so that the sum of task losses of all the scale factors sampled in the distribution $p\left(r\right)$ is the smallest.
Finally, we use the trained network for the inference. Suppose that the scale factor of the current task is r, the length of the input LR image corresponding to the current task is L, and the width is W, so the length of the SR image is ⌊L×r⌋, and the width is ⌊W×r⌋. For each pixel in the SR image, the convolution weight prediction network generates a convolution kernel matching its regularized pattern according to Equation (7). Then the generated convolution kernel is used to map the LR features of the corresponding positions to RGB values according to Equation (9), and finally, the SR image is formed. Figure 2 is an example of SR images generated with scale factors of 1.6, 2.2, 2.8, 3.4, and 4.0, respectively.

### 2.3. Justification of the Proposed Dynamic Convolution Kernel Generation Method

To demonstrate the various convolution kernels generated according to different image content, an experiment is conducted as follows.

Assuming that the scale factor r is 2, for the pixel $X\left(i^{\prime },j^{\prime }\right)$ in the low-resolution image $I^{LR}$, we can generate four convolution kernels $W_{2i^{\prime },2j^{\prime }}$, $W_{2i^{\prime }+1,2j^{\prime }}$, $W_{2i^{\prime },2j^{\prime }+1}$, $W_{2i^{\prime }+1,2j^{\prime }+1}$. We define these convolution kernels as a convolution kernel group $G_{i^{\prime },j^{\prime }}$ on this same pixel location, which corresponds to $G_{i^{\prime },j^{\prime }}^{1}$, $G_{i^{\prime },j^{\prime }}^{2}$, $G_{i^{\prime },j^{\prime }}^{3}$, $G_{i^{\prime },j^{\prime }}^{4}$, and the variation of the convolution kernel group $G_{i^{\prime },j^{\prime }}$ at pixel point $X\left(i^{\prime },j^{\prime }\right)$ in $I^{LR}$ is defined as

(14) $$C_{i^{\prime },j^{\prime }}=\sum_{p,q\in \left\{-1,0,1\right\}}\sum_{x\in \left\{1,2,3,4\right\}}D\left(G_{i^{\prime },j^{\prime }}^{x},G_{i^{\prime }+p,j^{\prime }+q}^{x}\right)$$

where the $C_{i^{\prime },j^{\prime }}$ is the variation of the convolution kernel group $G_{i^{\prime },j^{\prime }}$ at the pixel point $X\left(i^{\prime },j^{\prime }\right)$ in $I^{LR}$, and D() is the function of calculating the variation between the two convolution kernel groups and defined as

(15) $$D\left(G_{i^{\prime },j^{\prime }}^{x},G_{i^{\prime }+p,j^{\prime }+q}^{x}\right)=\sum_{m\in G_{i^{\prime },j^{\prime }}^{x},n\in G_{i^{\prime }+p,j^{\prime }+q}^{x}}abs\left(m-n\right)$$

where abs() is an absolute value function, and m, n are different values at corresponding positions in two different convolution kernels.

In our experiment, we use the test image 253,027 from the B100 dataset [19], the img59 image from the Urban100 dataset [20], and the YumeiroCooking image from the Manga109 dataset [21] as the test images. Then, we apply Equation (10) on these images to obtain the variation value of the convolution kernel group at each position, and then normalize the values to be a range of [0, 255]. These values are visualized as color images using COLORMAP_JET in OpenCV.

**Figure 3:**
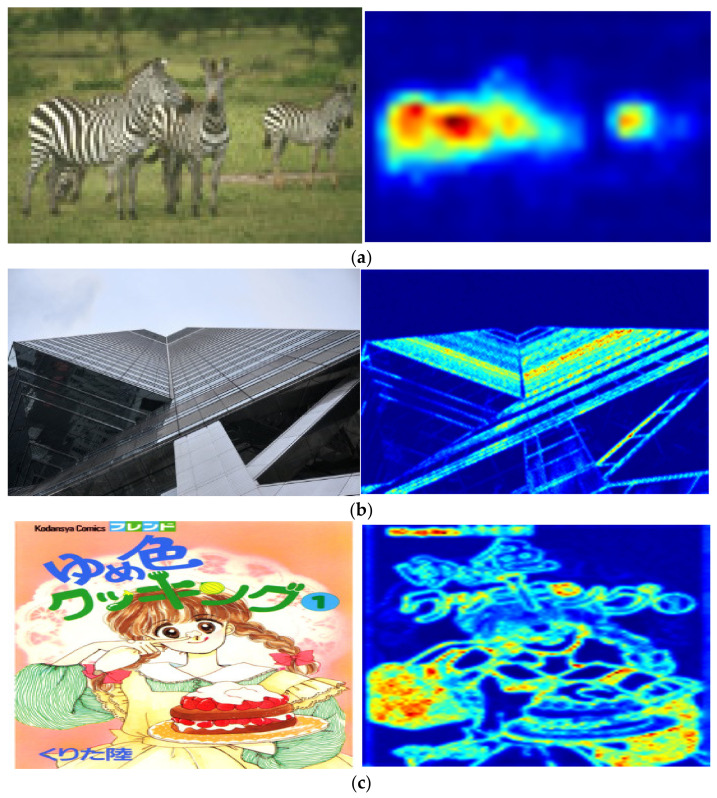
Visualization of dynamic convolution weights that are generated by our proposed approach. The first column presents the original test images, and the second column presents the visualized convolution weight variation values calculated using Equation (10). (**a**) 253027 from B100 dataset [19]. (**b**) img59 from Urban100 dataset [20]. (**c**) YumeiroCooking from Manga109 dataset [21].

As seen from Figure 3, we can find that in the grassland, sky, and large-area color blocks, where the content changes slowly and the regularization pattern is relatively simple, the change variation of the convolution kernel group is very small. The convolution kernel group of these pixel points is very similar to the convolution kernel groups of their neighbor pixels. On the contrary, the zebra patterns, clothing patterns, and architectural textures change drastically. The regularized pattern yields rich information, the convolution kernel group changes much. The regularized pattern guides the generation of the convolution kernel, which prompts the convolution weight prediction network to generate the optimal convolution kernel.

### 2.4. Proposed Image Super-Resolution Approach

**Figure 4:**
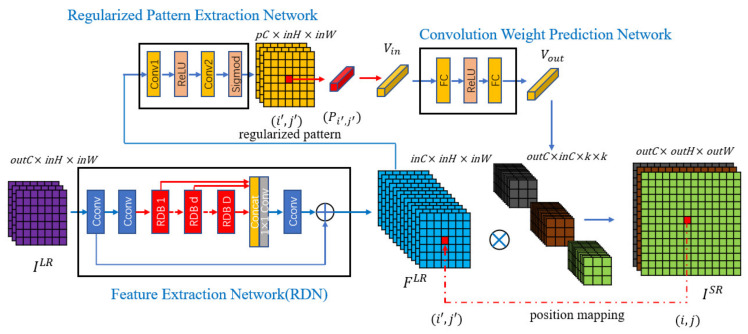
An overview of the network structure of the proposed SR approach.

An overview of the proposed network structure is shown in Figure 4. It contains three parts: (i) feature extraction network, (ii) regularized pattern extraction network, and (iii) convolution weight prediction network. We name our network as *Regularized Pattern Based-RDN* (RPB-RDN) since we chose RDN [10] as the first-part feature extraction network, which has been used also in Meta-RDN [16] and LIIF-RDN [17]. The second part regularized pattern extraction network and the third part convolution weight prediction network are presented as follows, respectively.

The regularized pattern extraction network consists of two convolutional layers, a ReLU activation function layer, and a Sigmoid activation function layer. Both the numbers of input and output channels of the *Conv1* layer are inC, and the Relu activation function layer is used to perform nonlinear mapping on the LR feature $F^{LR}$. The number of input channels of the *Conv2* layer is inC, and the number of output channels is outC, so that the final regularized pattern has a suitable number of channels. Finally, the Sigmoid activation function layer maps the regularized pattern to [0, 1] so that it has the same value range as the position and scale information.

The convolution weight prediction network consists of two full connection layers and a ReLU activation function layer. We concatenate the regularized pattern vector of the current position and the position and scale information to get the vector $V_{in}$ as the input of the first full connection layer. In our network, the dimensions of the regularized pattern vector $P_{i^{\prime },j^{\prime }}$ and the position and scale information vector $M_{i,j}$ are both 3, so the dimension of the vector $V_{in}$ is 6. Considering that the output vector dimension of the entire convolution weight prediction network is outC×inC×k×k, we set the number of output units of the first full connection layer to 256 for the diversity of the output of the entire convolution weight prediction network while ensuring speed. Therefore, the number of input units of the second full connection layer is 256, and the output of that is a vector $V_{out}$ whose dimension is outC×inC×k×k. Then we transform $V_{out}$ into a group of convolution kernels. The number of convolution kernels is the same number of SR image gray channels outC, and the parameter number of each convolution kernel is inC×k×k. This convolution weight prediction network is expressed as

(16) $$V_{out}=W_{2}\left(\sigma \left(W_{1}V_{in}+b_{1}\right)\right)+b_{2}$$

where $W_{i}$ is the weight of the *i*th fully-connected layer, $b_{i}$ is the bias of the *i*th fully connected layer and *σ*() is the Relu activation function.

## 3. Experimental Results

To evaluate the performance of the proposed RPB-RDN network and its various proposed components, including the proposed regularized pattern extraction network and the convolution weight prediction method, extensive experimental results are provided in this section, including the comparison between RPB-RDN and other SOTA methods.

### 3.1. Experimental Setup

In this paper, the high-resolution image set DIV2K is used. There are a total of 1000 images in DIV2K, 800 images for training, 100 images for verification, and 100 images for testing. All experimental models are trained with a DIV2K training image set. For testing, five standard benchmark data sets are used, including Set5 [22], Set14 [23], B100 [19], Urban100 [20], and Manga109 [21]. The PSNR and SSIM performance metrics are used to evaluate the results of image super-resolution reconstruction. All performance metrics are calculated on the Y channel of the YCbCr color space of the image. Given two images, the detailed formulas of PSNR and SSIM [24] are provided as

(17) $$PSNR=10\times log_{10}(\frac{MaxV}{MSE})$$

(18) $$SSIM\left(x,y\right)=\frac{\left(2\mu_{x}\mu_{y}+c_{1}\right)\left(2\sigma_{xy}+c_{2}\right)}{\left(\mu_{x}^{2}+\mu_{y}^{2}+c_{1}\right)\left(\sigma_{x}^{2}+\sigma_{y}^{2}+c_{2}\right)}$$

where MaxV is the maximum intensity value that image pixels can take, MSE is the mean square error between the two images, $\mu_{x}$ is the average intensity value of the image x, $\mu_{y}$ is the average intensity value of the image *y*, $\sigma_{x}^{2}$ is the variance of image *x*, $\sigma_{y}^{2}$ is the variance of image y, $\sigma_{xy}$ is the covariance of image *x* and image *y*, *c*_1_ and *c*_2_ are constants used to maintain stability [24].

### 3.2. Implementation Details

We use the L1 loss function to train the network. During the network training process, 8 low-resolution image patches with a size of 50 × 50 are randomly selected as a batch input. We increase the number of patches by flipping horizontally or vertically and randomly rotating 90°. The optimizer is Adam, and the learning rate is initialized to 0.0001, which is reduced by every 400 epochs. All experiments are run in parallel on 2 GPUs. The training scale factor varies from 1 to 4, the step size is 0.1, and the distribution of the scale factors is uniform. Each image patch in a batch has the same scale factor. The dimension of the regularized pattern vector $P_{i^{\prime },j^{\prime }}$ is set to 3, which can speed up the matching efficiency and improve the reconstruction effect.

### 3.3. Performance Evaluation on the Proposed Regularized Pattern Extraction Method

To study the impact of the regularized pattern extraction method, an experiment is conducted to compare two network structures as follows. The first one (denoted as ‘*baseline model*’) is a single-layer convolutional network, which only performs a limited linear transformation on LR features. The second one is our proposed network. Since the proposed model uses the Sigmoid activation function at the end of the network, the regularized pattern is the same as the value range of the position and scale information, which can help to identify the relationship between input and output and speeds up the network convergence. Table 1 shows that the proposed model achieves better results in X2, X3, and X4 SR tasks in the three data sets of B100, Urban100, and Manga109, with an average increase of 0.04 dB in PSNR and 0.0005 in SSIM compared with the baseline model.

### 3.4. Performance Evaluation on the Proposed Convolution Weight Prediction Method

To verify the effectiveness of the convolution weight prediction method based on regularized pattern, an experiment is conducted using the benchmark dataset B100 [19] with scale factors ranging from 1.1 to 4.0 and a step length of 0.1 using Meta-RDN [16], and our RPB-RDN model respectively. As shown in Table 2, the proposed model, which integrates the convolution weight prediction method based on the regularized pattern, achieves better results than Meta-RDN [16] in all tasks with different scale factors. In a total of thirty tasks, RPB-RDN improves PSNR by 0.06 dB on average over Meta-RDN [16].

### 3.5. Performance Evaluation on the Inference Time

In this experiment, we compare the running time of RDN [10], Meta-RDN [16], LIIF-RDN [17], and our RPB-RDN using Xeon4210 and NVIDIA 2080Ti. We choose the B100 [19] as the test dataset and take the image pre-processing time out of consideration. The experimental results are shown in Table 3. The meta-upsampling module in RPB-RDN is more time-consuming than the sub-pixel convolutional layer in RDN [10], so the overall time-consumption of RPB-RDN is longer than RDN [10]. Compared with Meta-RDN [16], RPB-RDN adds a regularized pattern extraction network so that the overall time consumption has increased, but the difference is not large. LIIF-RDN [17] uses a multi-layer perceptron that is more time-consuming than convolutional layers, so the overall time-consuming of LIIF-RDN [17] is longer than RPB-RDN.

### 3.6. The Superior of the Proposed Method in Texture Reconstruction

We propose a texture dataset Texture, which crops the central part of images from five benchmark datasets Set5 [22], Set14 [23], B100 [19], Urban100 [20], and Manga109 [21]. The size of the cropped image is 1/16 of the original image. The foreground part in the center of the image generally has richer textures than the background part, and it is more difficult to restore. Comparing the SR results of the texture dataset Texture can further explore the texture image restoration ability of various methods.

We use Meta-RDN [16], LIIF-RDN [17], and RPB-RDN to perform the X2, X3, and X4 super-resolution reconstruction tasks on the proposed texture dataset Texture. The experimental results are shown in Table 4. RPB-RDN achieves better results than Meta-RDN [16] and LIIF-RDN [17] on all scales, which proves the superiority of the proposed content-adaptive convolution kernel generation methods for texture restoration. On the PSNR metric, RPB-RDN has an average improvement of 0.13 dB and 0.14 dB over Meta-RDN [16] and LIIF-RDN [17]. On the SSIM metrics, RPB-RDN has an average improvement of 0.0007 and 0.0008 over Meta-RDN [16] and LIIF-RDN [17].

### 3.7. Performance Comparison with Other SOTA Methods

We use the proposed network model to perform X2, X3, and X4 super-resolution reconstruction on benchmark datasets Set5 [22], Set14 [23], B100 [19], Urban100 [20], and Manga109 [21] respectively, and compare the results with RDN [10], Meta-RDN [16], and LIIF-RDN [17]. As seen from Table 5, we can see that our method achieves better results than these state-of-the-art methods in most of the reconstruction tasks. On all fifteen tasks, our method outperforms RDN [10], Meta-RDN [16], and LIIF-RDN [17] by an average of 0.10 dB, 0.11 dB, and 0.11 dB in PSNR, and 0.0007, 0.0003, and 0.0005 in SSIM, respectively. Especially in the high-resolution benchmark datasets Urban100 [20] and Manga109 [21], where the images have more richer details, our proposed model can achieve an improvement of 0.22 dB, 0.21 dB, and 0.21 dB in PSNR, and 0.0016, 0.0007, and 0.0010 in SSIM than RDN [10], Meta-RDN [16], and LIIF-RDN [17], respectively.

### 3.8. Qualitative Results

**Figure 5:**
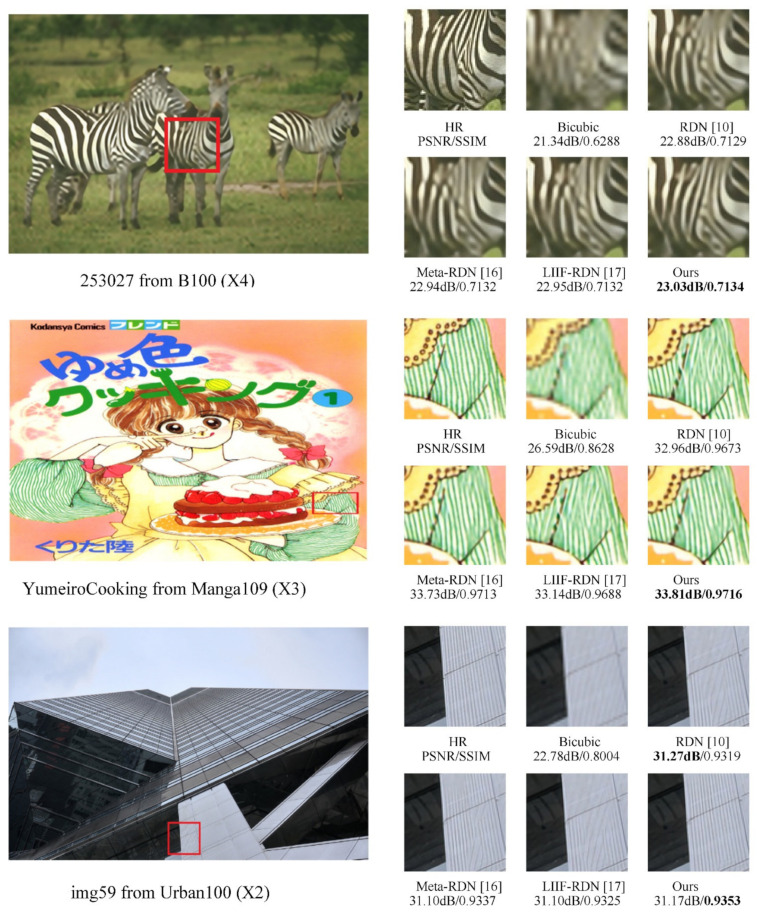
Qualitative performance comparison of various SR approaches.

Finally, we compare the SR images generated by our RPB-RDN with those generated by Bicubic, RDN [10], Meta-RDN [16], and LIIF-RDN [17]. As seen in Figure 5, it can be found that our method can recover textures that are recovered wrongly by other methods, especially in zebra patterns, patterns on clothes, and lines of buildings. Owing to our regularized pattern-based convolution kernel generation method, pixels in the different regularized patterns will generate different convolution kernels to match the regularized pattern so that the generated SR image and HR image have stronger texture consistency.

## 4. Conclusions

In this paper, we propose a dynamic kernel generation method based on the regularized pattern for super-resolution image reconstruction. It can generate convolution kernels for different pixels that match their regularized pattern so that the generated SR images have stronger texture consistency with HR images. Experiments show that our proposed method achieves better performance than other state-of-the-art approaches.

## Figures and Tables

**Table 1:** PSNR (dB) and SSIM performance on proposed regularized pattern extraction network. The best performance is highlighted in the bold format.

Methods	Metric	B100 [19]			Urban100 [20]			Manga109 [21]		
		X2	X3	X4	X2	X3	X4	X2	X3	X4
Baseline model	PSNR SSIM	32.34 0.9011	29.27 0.8089	27.75 0.7417	33.00 0.9359	28.90 0.8668	26.68 0.8042	39.31 0.9781	34.41 0.9491	31.36 0.9173
RPB-RDN (Ours)	PSNR SSIM	**32.36** **0.9014**	**29.30** **0.8095**	**27.76** **0.7421**	**33.04** **0.9363**	**28.95** **0.8677**	**26.73** **0.8054**	**39.35** **0.9782**	**34.46** **0.9494**	**31.39** **0.9177**

**Table 2:** PSNR (dB) performance evaluation on the proposed convolution weight prediction method using the B100 dataset [19]. The best performance is highlighted in the bold format.

Methods	X1.1	X1.2	X1.3	X1.4	X1.5	X1.6	X1.7	X1.8	X1.9	X2.0
Bicubic	36.56	35.01	33.84	32.93	32.14	31.49	30.90	30.38	29.97	29.55
Meta-RDN [16]	42.82	40.04	38.28	36.95	**35.86**	34.90	34.13	33.45	**32.86**	32.35
Ours	**43.03**	**40.11**	**38.34**	**36.96**	**35.86**	**34.91**	**34.14**	**33.46**	**32.86**	**32.36**
Methods	X2.1	X2.2	X2.3	X2.4	X2.5	X2.6	X2.7	X2.8	X2.9	X3.0
Bicubic	29.18	28.87	28.57	28.31	28.13	27.89	27.66	27.51	27.31	27.19
Meta-RDN [16]	31.82	31.41	31.06	30.62	30.45	30.13	29.82	29.67	29.40	**29.30**
Ours	**31.88**	**31.45**	**31.07**	**30.75**	**30.48**	**30.17**	**29.95**	**29.72**	**29.49**	**29.30**
Methods	X3.1	X3.2	X3.3	X3.4	X3.5	X3.6	X3.7	X3.8	X3.9	X4.0
Bicubic	26.98	26.89	26.59	26.60	26.42	26.35	26.15	26.07	26.01	25.96
Meta-RDN [16]	28.87	28.79	28.68	28.54	28.32	**28.27**	28.04	27.92	27.82	27.75
Ours	**29.09**	**28.90**	**28.73**	**28.57**	**28.42**	**28.27**	**28.15**	**28.01**	**27.88**	**27.76**

**Table 3:** The running time in B100 of various SR approaches (ms). The best performance is highlighted in the bold format.

Methods	X2	X3	X4
RDN	**12.8**	**12.9**	**13.0**
Meta-RDN [16]	14.4	14.8	16.4
LIIF-RDN [17]	21.3	22.7	24.9
RPB-RDN (Ours)	15.1	15.3	16.5

**Table 4:** The PSNR (dB) and SSIM performance in texture dataset *Texture*. The best performance is highlighted in the bold format.

Methods	Metric	X2	X3	X4
Meta-RDN [16]	PSNR(dB)/SSIM	34.22/0.9312	30.30/0.8601	28.08/0.7976
LIIF-RDN [17]		34.19/0.9309	30.31/0.8601	28.09/0.7975
RPB-RDN (Ours)		**34.38/0.9318**	**30.45/0.8611**	**28.17/0.7981**

**Table 5:** The PSNR (dB) and SSIM performance comparison of various SR approaches. The best performance is highlighted in the bold format.

Dataset	The PSNR (dB) Performance					
	Scale Factor	Bicubic	RDN [10]	Meta-RDN [16]	LIIF-RDN [17]	Ours
Set5 [22]	X2 X3 X4	33.66 30.39 28.42	**38.24** 34.71 32.47	38.22 34.63 32.38	38.17 34.68 **32.50**	38.23 **34.74** **32.50**
Set14 [23]	X2 X3 X4	30.24 27.55 26.00	34.01 **30.57** 28.81	34.04 30.55 28.84	33.97 30.53 28.80	**34.05** 30.56 **28.86**
B100 [19]	X2 X3 X4	29.56 27.21 25.96	32.34 29.26 27.72	32.35 29.30 27.75	32.32 29.26 27.74	**32.36** **29.30** **27.76**
Urban100 [20]	X2 X3 X4	26.88 24.46 23.14	32.89 28.80 26.61	32.92 28.82 26.55	32.87 28.82 26.68	**33.04** **28.95** **26.73**
Manga109 [21]	X2 X3 X4	30.80 26.95 24.89	39.18 34.13 31.00	39.18 34.14 31.03	39.01 34.13 31.18	**39.35** **34.46** **31.39**
**Dataset**	**The SSIM Performance**					
	**Scale factor**	**Bicubic**	**RDN** [10]	**Meta-RDN** [16]	**LIIF-RDN** [17]	**Ours**
Set5 [22]	X2 X3 X4	0.9299 0.8682 0.8104	**0.9614** 0.9296 **0.8990**	0.9611 **0.9298** 0.8989	0.9610 0.9293 0.8986	0.9611 **0.9298** **0.8990**
Set14 [23]	X2 X3 X4	0.8688 0.7742 0.7027	0.9212 0.8468 0.7871	0.9213 0.8466 0.7872	0.9208 **0.8470** 0.7876	**0.9214** **0.8470** **0.7881**
B100 [19]	X2 X3 X4	0.8431 0.7385 0.6675	0.9017 0.8093 0.7419	**0.9019** **0.8096** **0.7423**	0.9010 **0.8096** 0.7422	0.9014 0.8095 0.7421
Urban100 [20]	X2 X3 X4	0.8403 0.7349 0.6577	0.9353 0.8653 0.8028	0.9361 0.8674 **0.8054**	0.9350 0.8662 0.8040	**0.9363** **0.8677** **0.8054**
Manga109 [21]	X2 X3 X4	0.9339 0.8556 0.7866	0.9780 0.9484 0.9151	**0.9782** 0.9483 0.9154	0.9780 0.9487 0.9170	**0.9782** **0.9494** **0.9177**

## Data Availability

Not applicable.

## References

[1] Jian M., Lam K. (2015). Simultaneous Hallucination and Recognition of Low-Resolution Faces Based on Singular Value Decomposition. IEEE Trans. Circuits Syst. Video Technol..

[2] Jian M., Cui C., Nie X., Zhang H., Nie L., Yin Y. (2019). Multi-view face hallucination using SVD and a mapping model. Inf. Sci..

[3] Dong C., Loy C.C., He K., Tang X. (2014). Learning a deep convolutional network for image super-resolution. Proceedings of the ECCV 2014.

[4] Kim J., Lee J.K., Lee K.M. (2016). Accurate image super-resolution using very deep convolutional networks. Proceedings of the IEEE Conference on Computer Vision and Pattern Recognition.

[5] Kim J., Lee J.K., Lee K.M. (2016). Deeply-recursive convolutional network for image super-resolution. Proceedings of the 2016 IEEE Conference on Computer Vision and Pattern Recognition (CVPR).

[6] Tai Y., Yang J., Liu X. (2017). Image super-resolution via deep recursive residual network. Proceedings of the IEEE Conference on Computer Vision and Pattern Recognition (CVPR).

[7] Tai Y., Yang J., Liu X., Xu C. (2017). Memnet: A persistent memory network for image restoration. Proceedings of the 2017 IEEE International Conference on Computer Vision (ICCV).

[8] Shi W., Caballero J., Huszar F. (2016). Real-time single image and video super-resolution using an efficient sub-pixel convolutional neural network. Proceedings of the 2016 IEEE Conference on Computer Vision and Pattern Recognition (CVPR).

[9] Lim B., Son S., Kim H. (2017). Enhanced deep residual networks for single image super-resolution. Proceedings of the IEEE Conference on Computer Vision and Pattern Recognition Workshops (CVPRW).

[10] Zhang Y., Tian Y., Kong Y., Zhong B., Fu Y. (2018). Residual dense network for image super-resolution. Proceedings of the IEEE Conference on Computer Vision and Pattern Recognition (CVPR), Salt Lake City.

[11] Liu J., Zhang W., Tang Y., Tang J., Wu G. (2020). Residual Feature Aggregation Network for Image Super-Resolution. Proceedings of the IEEE Conference on Computer Vision and Pattern Recognition (CVPR).

[12] Dai T., Cai J., Zhang Y., Xia S., Zhang L. (2019). Second-order Attention Network for Single Image Super-Resolution. Proceedings of the IEEE Conference on Computer Vision and Pattern Recognition (CVPR).

[13] Li L., Feng H., Zheng B., Ma L., Tian J. (2021). DID: A nested dense in dense structure with variable local dense blocks for super-resolution image reconstruction. Proceedings of the 25th International Conference on Pattern Recognition (ICPR).

[14] Hospedales T., Antoniou A., Micaelli P., Storkey A. (2020). Meta-Learning in Neural Networks: A Survey. arXiv.

[15] Ha D., Dai A., Le Q.V. HyperNetworks. Proceedings of the International Conference on Learning Representations (ICLR).

[16] Hu X., Mu H., Zhang X., Wang Z., Tan T., Sun J. (2019). Meta-SR: A Magnification-Arbitrary Network for Super-Resolution. Proceedings of the IEEE Conference on Computer Vision and Pattern Recognition (CVPR).

[17] Chen Y., Liu S., Wang X. Learning Continuous Image Representation with Local Implicit Image Function. Proceedings of the IEEE Conference on Computer Vision and Pattern Recognition (CVPR).

[18] Li D., Hu J., Wang C., Li X., She Q., Zhu L., Zhang T., Chen Q. Involution: Inverting the Inherence of Convolution for Visual Recognition. Proceedings of the IEEE Conference on Computer Vision and Pattern Recognition (CVPR).

[19] Martin D., Fowlkes C., Tal D., Malik J. (2001). A database of human segmented natural images and its application to evaluating segmentation algorithms and measuring ecological statistics. Proceedings of the Eighth IEEE International Conference on Computer Vision. ICCV 2001.

[20] Huang J.-B., Singh A., Ahuja N. (2015). Single image super-resolution from transformed self-exemplars. Proceedings of the 2015 IEEE Conference on Computer Vision and Pattern Recognition (CVPR).

[21] Matsui Y., Ito K., Aramaki Y., Fujimoto A., Ogawa T., Yamasaki T., Aizawa K. (2017). Sketch-based manga retrieval using manga109 dataset. Multimed. Tools Appl..

[22] Bevilacqua M., Roumy A., Guillemot C., Morel A. Low-complexity single-image super-resolution based on nonnegative neighbor embedding. Proceedings of the British Machine Vision Conference (BMVC).

[23] Zeyde R., Elad M., Protter M. (2010). On single image scale-up using sparse representations. Proceedings of the International Conference on Curves and Surfaces.

[24] Wang Z., Bovik A.C., Sheikh H.R., Simoncelli E.P. (2004). Image quality assessment: From error visibility to structural similarity. IEEE Trans. Image Process..

